# Classification Evolution and Epitope Prediction of the Porcine Epidemic Diarrhea Virus (PEDV) Spike Protein in Thailand (2008–2024): Updated Insights for Preventive Strategies

**DOI:** 10.3390/ani16152314

**Published:** 2026-07-27

**Authors:** Christopher James Stott, Tanakamol Mahawan, Pablo Piñeyro, Hongyao Lin, Angkana Tantituvanont, Dachrit Nilubol

**Affiliations:** 1 Akkhararatchakumari Veterinary College, Walailak University, Nakhon Si Thammarat 80160, Thailand; christopher.st@wu.ac.th (C.J.S.);; 2 Research Center in One Health, Walailak University, Nakhon Si Thammarat 80160, Thailand; 3 Department of Veterinary Diagnostic and Production Animal Medicine, College of Veterinary Medicine, Iowa State University, Ames, IA 50011-1134, USA; 4 MSD Animal Health Innovation Pte Ltd., Singapore 718847, Singapore; 5 Department of Pharmaceutics and Industrial Pharmacy, Faculty of Pharmaceutical Sciences, Chulalongkorn University, Bangkok 10330, Thailand; 6 Center of Excellence in Swine Viral Evolution and Vaccine Development, Department of Veterinary Microbiology, Faculty of Veterinary Science, Chulalongkorn University, Bangkok 10330, Thailand

**Keywords:** swine enteric coronavirus, phylogenetic, preventive medicine, vaccine, diarrhea, computational analysis

## Abstract

This study analyzed Porcine epidemic diarrhea virus (PEDV) spike protein sequences and structures in Thailand from 2008 to 2024 to provide predicted structural templates that could inform regional vaccine selection and planned exposure frameworks. Using an in-silico approach, the researchers reduced sequence redundancy via CD-HIT (v4.8.1), established evolutionary lineages with BEAST (v1.10.4), and reconstructed protein structures using SWISS-MODEL. Structural comparisons and clustering were performed using DALI Z-scores and DBSCAN (v1.2.2), while Discotope 3 (v3.0) and ElliPro mapped B-cell epitope landscapes against a G1 reference strain. The results revealed a major lineage shift from G2a to G2b strains around 2017, with the spike proteins categorized into 14 subtypes and 6 eigenvalue clusters. Notably, minor amino acid substitutions altered properties such as hydrophobicity without disrupting the core structure, and certain deletions caused minimal structural deviations, indicating that sequence data or predicted structures alone do not fully dictate viral virulence or immunogenicity. Furthermore, primitive TH2 strains shared evolutionary links with G1 or US-InDel strains despite their G2 classification, identifying Cluster 1 as a potential ancestral structural type. In conclusion, this updated analysis provides crucial baseline data to optimize regional PEDV preventative measures, though further rigorous structural investigations are needed to definitively link specific spike alterations to virulence and host immune response.

## Simple Summary

Porcine epidemic diarrhea virus (PEDV) continues to cause severe watery diarrhea and devastating piglet production losses worldwide. To map the long-term evolutionary trends of the virus in Thailand, this study evaluated clinical samples collected over a 16-year period (2008–2024) using a comprehensive molecular and bioinformatic pipeline. By analyzing the predicted full-length spike (S) protein structures modeled from nucleotide sequences, we tracked critical mutational shifts and predicted antigenic variation within major neutralizing domains (including COE, SS2, SS6, and 2C10) that may influence antibody recognition. The findings reveal the emergence of unique insertion/deletion (InDel) strains and establish that primitive Thai strains (Cluster 1) likely represent the original structural template of PEDV in the region prior to subsequent divergence into G1 and G2b-related lineages. Ultimately, this framework bridges genomic evolution with structural biology, providing essential data to guide vaccine selection, optimize planned exposure protocols, and update swine biosecurity strategies.

## 1. Introduction

Porcine epidemic diarrhea virus (PEDV) is a member of the subgenus *Pedacovirus*, genus *Alphacoronavirus*, family *Coronaviridae*, and order *Nidovirales*. It causes porcine epidemic diarrhea (PED), a highly contagious enteric disease in swine. While pigs of all ages are susceptible, the disease is particularly lethal in neonatal piglets, frequently resulting in high mortality. Affected pigs typically present with severe watery diarrhea, vomiting, and acute dehydration, which can be fatal [1,2,3]. The virus is primarily transmitted via the fecal-oral route, with replication occurring within the intestinal epithelial cells. Detection of PEDV in sow milk and alveolar macrophages suggests potential vertical and respiratory transmission [4]. The severity of PEDV infection is typically evaluated by histopathological examination of villous atrophy. The virus damages the villous epithelium, leading to progressive shortening and reduced nutrient absorption, which results in worsened clinical signs [5].

PEDV possesses an enveloped, positive-sense, single-stranded RNA genome approximately 28 kb in length, comprising seven open reading frames (ORFs): ORF1a, ORF1b, spike (S), ORF3, envelope (E), membrane (M), and nucleocapsid (N) genes [6,7]. The virus is classified into two major genogroups, G1 and G2, with G2 further subdivided based on spike gene sequences into at least G2a, G2b, S-InDel (insertion/deletion; G2c or sometimes G1b), and recombinant variants. Genogroup G2a is endemic to Asia and has been detected in countries including China, South Korea, and Thailand, while G2b strains are responsible for global outbreaks [8,9].

The spike protein is critical for viral entry, as it mediates binding to host cell receptors. It consists of two subunits: S1 (amino acids 1–725) and S2 (amino acids 726–1383) [8,10]. Due to its high variability and antigenic significance, the S protein is the primary focus of genetic and immunological research. Four key neutralizing epitopes have been identified on the S protein: CO-26K-equivalent epitope (COE epitope; amino acids 499–638), SS2 (748–755), SS6 (764–771), and 2C10 (1368–1374), in addition to a recently reported novel neutralizing epitope, mAbA3 (amino acids 249–260) [10,11,12,13]. Additionally, a novel enhancing epitope, mAbG3, was recently identified [14], encompassing regions M3S-0 (176–187) and M4S1-BCD (643–654). Recent studies in China have highlighted high sequence variability within the signal peptide (SP) and the S1 N-terminal domain (S1-NTD) among circulating strains [10]. Based on structural comparisons with HCoV-NL63, the S1 subunit can be further segmented into the SP (1–18), subdomain 0 (19–219), subdomain A (220–509), subdomain B (510–639), and subdomain CD (640–728) [15].

Previous reports have demonstrated that substitutions or recombinations within the spike gene can significantly alter viral virulence, leading to either attenuation or increased pathogenicity. For instance, specific substitutions, insertions, or deletions at positions 56 (GENQ motif) and 155–156 (DG motif) of the spike protein amino acid sequence are associated with the transition from the G1 to the G2 genogroup. Furthermore, specific motif changes within the spike protein are recognized as key factors in the attenuation of PEDV strains [16].

Thailand first recognized PED in 1995, and the virus was confirmed as a G2a strain by 2007. Since then, recurrent outbreaks have been predominantly linked to G2a lineages, including G2a-TH1 and G2a-TH2. While the situation remained relatively stable with only sporadic outbreaks, a recombination event between G2a-TH1 and G2a-TH2 (designated G2a-TH3) was discovered in 2011. However, the epidemiological situation changed significantly in 2014 due to a large-scale outbreak caused by G2a-TS1 (a TH1 variant with a unique insertion) [9]. This was followed by the discovery of the first China-like G2b strain in Thailand (G2b-TH4), which shared a common ancestor with contemporaneous Vietnamese strains. Additionally, the first G1 strain (G1a-TH5) was identified in eastern Thailand within herds previously vaccinated against G1 [17].

During the 2014 large-scale outbreak, the US-like G2b strain (G2b-TH6) was also reported; however, it appeared only once and has not been detected since, suggesting limited adaptation or transmission within Thailand. After the infections caused by the G2a-TH1 insertion variants (G2a-TS1) subsided, the emergence of China-like G2b strains in sporadic outbreaks posed new challenges. Notably, recombination between G2a-TH1 and G2b-TH4, as well as mutations in G2b-TH4 leading to spike gene insertions, have further complicated disease control efforts.

Given the role of the PEDV spike protein in host interaction, immune recognition, and conformational epitope presentation, structural analysis may provide additional insight into the consequences of spike sequence variation. Such information is not always evident from primary sequence comparison alone. Therefore, DALI-based structural comparison was used in this study to complement amino acid-based phylogenetic analysis and B-cell epitope prediction [18].

Planned exposure to field strains has been implemented across multiple regions as a strategy for managing PEDV outbreaks. Specifically, G2a strains have been utilized within stable herds in western Thailand, whereas G2b strains have been deployed in the eastern region to control local variants and recombinant strains. Despite ongoing surveillance and an increasing understanding of PEDV genogroups, comprehensive and integrated analyses encompassing sequence evolution, structural variation, and epitope profile shifts in Thai PEDV spike proteins from 2016 to 2024 remain limited. Adopting such an integrated approach is useful for mapping the adaptive landscape of the virus in Thailand and for offering baseline structural insights for future vaccine evaluation. Consequently, continuous monitoring of spike protein mutations provides foundational data to guide the targeted experimental validation of next-generation vaccine candidates.

This study aims to characterize the classification of PEDV spike proteins among Thai field strains by utilizing a comprehensive dataset of previously reported nucleotide sequences alongside an updated data from the Department of Veterinary Microbiology, Chulalongkorn University. We further investigate the molecular characteristics of these strains through an integrated approach involving amino acid-based phylogenetic analysis, protein structural modeling, and B-cell epitope mapping. By leveraging these analytical tools across a longitudinal dataset, this research seeks to provide insights into the structural evolution of the Thai PEDV spike protein. Ultimately, these findings are intended to inform enhanced preventive strategies and facilitate more effective vaccine design for the regional swine industry.

## 2. Materials and Methods

### 2.1. Data Collection and Preparation

A total of 315 PEDV nucleotide sequences were converted and utilized as the primary dataset. These included both published and unpublished full-length spike protein sequences obtained from previous research and samples submitted for PEDV identification to the Department of Veterinary Microbiology at Chulalongkorn University, Thailand, between 2008 and 2024. Inclusion criteria were strictly limited to field strain sequences containing the full coding region from the start to the stop codon. The dataset was subsequently subjected to CD-HIT (v4.8.1) [19] clustering based on amino acid sequence identity. This process began at 100% identity and decreased in 1% increments until the clusters demonstrated a clear epidemiological correlation with outbreak locations. This step ensured that the resulting dataset was representative of each protein group for subsequent downstream analyses.

### 2.2. Phylogenetic Inference and Molecular Evolution

To provide an initial overview of Thai PEDV clade distribution relative to global strains, the amino acid dataset was analyzed using FastTree (v2.1.11) [20] within the Geneious Prime 2025.1.2 software package. Subsequently, representative PEDV spike amino acid sequences, tip-dated by sampling year, were utilized to perform Bayesian phylogenetic inference using the BEAST package (v1.10.4) [21]. The analysis employed the BLOSUM62+G+I substitution model under an uncorrelated relaxed log-normal molecular clock. Markov Chain Monte Carlo (MCMC) chains were run with parameters logged every 10,000 states until all parameters reached convergence, defined by effective sample size (ESS) values > 200. Three distinct datasets were analyzed: (i) full-length spike amino acid sequences, (ii) COE region, and (iii) selected epitope partitions (mAbG3, mAbA3, SS6, and 2C10). The SS2 region was excluded from the analysis due to a lack of unique sequence identity across the sampled strains.

### 2.3. Structural Analysis and Classification of Predicted Protein Models

Representative PEDV spike amino acid sequences were subjected to tertiary structure prediction using SWISS-MODEL (http://swissmodel.expasy.org, accessed on 7 June 2025) [22]. The Pingtung 52 strain (PDB ID: 7W6M) was selected as the optimal template for G2 protein modeling based on superior Global Model Quality Estimation (GMQE) scores. To assess structural homology, the predicted models were compared using the DALI server (http://ekhidna2.biocenter.helsinki.fi/dali, accessed on 12 June 2025) [18], and a structural relationship tree was constructed based on pairwise Z-score similarities. Hierarchical clustering of these DALI Z-scores was performed to classify distinct protein structural archetypes. Furthermore, an eigenvalue plot derived from multidimensional correspondence analysis (MCA) was generated using RStudio (version 2025.05.1). Density-Based Spatial Clustering of Applications with Noise (DBSCAN; v1.2.2) [23] was subsequently applied with an epsilon (ε) parameter of 2.5; structural outliers identified as noise were excluded from the final clusters. Further analysis was conducted on the distinct InDel patterns of the TH4 lineage (TD4 and TS4), which exhibited greater variation and were associated with later outbreaks following the large-scale Thai outbreaks of 2014–2015. Advanced structural modeling for these variants was executed using AlphaFold3 (https://alphafoldserver.com, accessed on 10 July 2026) [24]. Concurrently, potential N-linked glycosylation sites across the representative PEDV spike protein sequences were predicted using the NetNGlyc 1.0 server [25]. Only sequons exhibiting the canonical Asn-Xaa-Ser/Thr (N-X-S/T, where X ≠ P) motif with a prediction score greater than 0.5 were considered structurally viable, the predicted tertiary structures were subsequently superimposed and compared in UCSF ChimeraX (v1.10) [26] using the MatchMaker tool to assess structural homology across strains.

### 2.4. Epitope Prediction

Discotope 3 [27] was used for B-cell epitope prediction, while ElliPro [28] was applied to identify both linear and discontinuous epitopes using all representative sequences. Epitope positions were mapped and standardized relative to the EAS1 (TH5; KR610991) reference strain. This strain was selected as a representative of the G1-like architecture, as its sequence length and genomic organization are identical to the prototype CV777 (NC_003436). Using EAS1 provided a stable baseline for identifying and characterizing the InDels observed in the contemporary Thai G2b field strains, ensuring a consistent amino acid numbering system across the dataset. Amino acid sites were analyzed based on several properties, including their aliphatic or hydrophobic, aromatic, hydrophilic, conformational, and charged characteristics. The discontinuous epitope patterns were then compared with those reported in a previous study [29] for further investigation, UCSF ChimeraX (v1.10) was used to visualize and map discontinuous epitopes.

## 3. Results

### 3.1. Global Phylogenetic Classification and Analysis of PEDV Amino Acid Sequences

A total of 3023 global PEDV sequences, including 315 from Thailand, were subjected to amino acid-based phylogenetic analysis. Detailed metadata (including GenBank accession numbers, collection years, and geographical origins for all sequences categorized by the genogroups defined in this study) are provided in Table S1.

**Figure 1:**
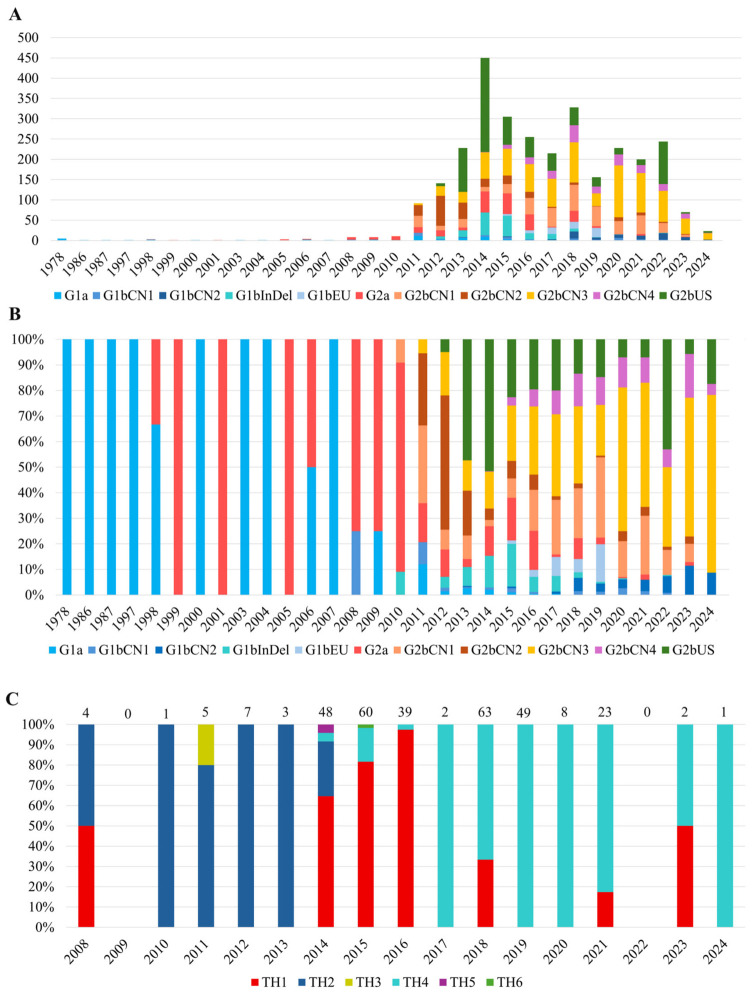
(**A**) Cumulative number of reported complete spike proteins available in GenBank, including the dataset used in this study. (**B**) Proportion of reported complete spike proteins in GenBank, including the dataset used in this study. (**C**) Proportion of investigated complete spike proteins from Thailand, classified into lineages TH1–TH6. Classification was based on amino acid sequences (from start to stop codon) using a FastTree phylogenetic tree constructed in this study.

**Figure 2:**
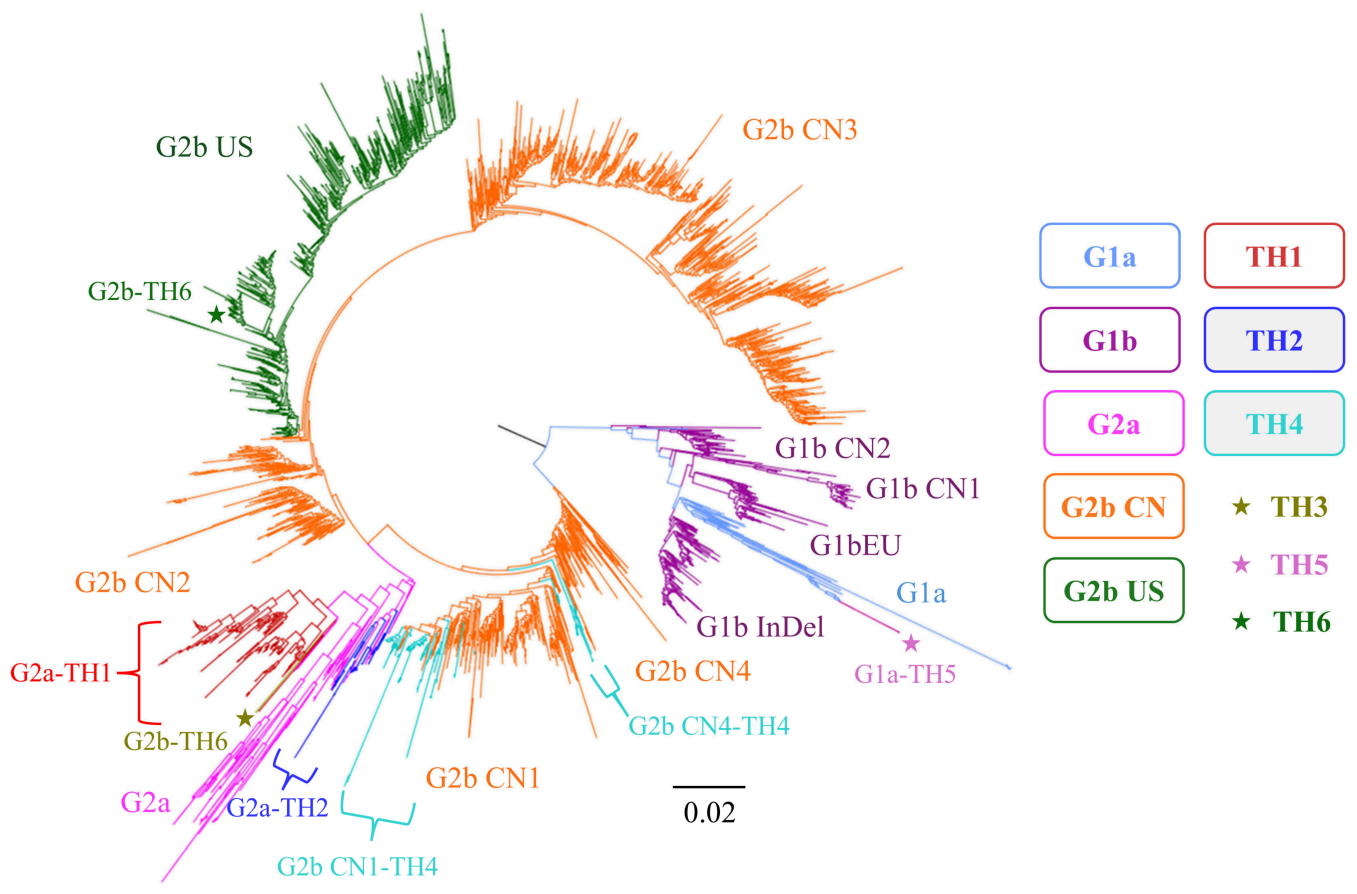
Phylogenetic tree of complete spike proteins (amino acid sequences from start to stop codon) constructed using FastTree. Classification follows previously reported groups: G1a, G1b (further classified into InDel, EU, CN1, and CN2 subgroups), G2a, and G2b (further classified into CN1, CN2, CN3, CN4, and US clades). Thailand PEDV spike proteins are highlighted using either colored branches or star markers (indicating invisible branches) according to their TH1–TH6 lineages.

Descriptive analysis of the dataset revealed the cumulative growth and proportions of spike protein sequences available in GenBank (Figure 1A,B). Within the Thai cohort, the investigated spike proteins were classified into lineages TH1–TH6 based on amino acid sequences from start to stop codon (Figure 1C). The resulting classification demonstrated a broad correlation with established nucleotide-based genogroups (Figure 2). This study identified two primary genogroups, G1 and G2. Within the G1 genogroup, the G1a clade was further subdivided into G1b-EU (Europe-like), G1b-CN1 (ancestral China G1b), G1b-CN2 (descendant China G1b), and G1b-InDel, the latter of which is also recognized as G2c or G2b-InDel in certain classification systems.

Notably, the complex and highly variable nature of Chinese PEDV was addressed through a high-resolution classification based on amino acid sequences. Within the G2b-CN lineage, multiple interspersed clusters were observed. G2b-CN4 formed a distinct, basal cluster positioned between the G2 and G1 genogroups, encompassing recombinant G2b-TH4 strains from Thailand. Following G2b-CN4, G2b-CN1 emerged as the earliest Chinese PEDV group within the G2 lineage and showed phylogenetic proximity to G2a. A large cluster of G2a strains was identified, predominantly from South Korea and Thailand, followed by the emergence of another distinct Chinese group, G2b-CN2. The phylogeny subsequently revealed a prominent cluster of G2b-US strains, ending with the G2b-CN3 cluster located at the periphery of the G2b lineage.

Thai strains, including TH1, TH2, TH4, and TH5, were distributed across various G2 subgroups. Specific examples such as G2a-TH3 and G2b-TH6 illustrated the complex genetic relationships and diverse circulation patterns in Thailand. In this study, the designations TS1 and TD1 were used for strains exhibiting insertions and deletions relative to TH1, respectively. The same naming convention was applied to TS2, TS4, and TD4 to facilitate clearer tracking of structural variants.

CD-HIT clustering at 99% identity revealed 61 clusters. Due to length variation among nine sequences, these divergent-length sequences were included for comparative analysis. All representative sequences were standardized to 70 representative sequences. Clustering at 99% identity corresponded more closely with outbreak regions than clustering at 100% (205 clusters) or 98% (37 clusters).

### 3.2. Protein Structure Classification

Due to the computational limitations of DALI, which allowed all-against-all comparisons for up to 64 structures, highly similar sequences were excluded using CD-HIT with a 98.3% identity threshold. This process resulted in the assignment of structure IDs SX1 through SX6. These structures were compared based on similarity clustering to identify initial protein groups, which were designated as groups A and B. Subsequently, all structures within these two groups were compared again for further refinement.

Among the sequences obtained from the field strains, we identified seven insertion sites. First, after position 55, insertions are widely used to classify strains as G2 PEDV (^56–58^GENQ); however, in our study, the insertion patterns included ^56–58^GENQ, ^56–58^GENH, ^56–58^ENH, and ^56–58^MNH. Second, an insertion after position 230 corresponds to the large-scale outbreak strains in Thailand during 2014–2015, featuring ^231^REY (with an N>T mutation at position 230; strains S17–18, S24–34, S63–64, and SX1), as well as a novel pattern found in a China-related strain, ^231^TTGR (strains S22, S57–58, and S62). Third, an insertion after position 303, ^304^DLKSF (S51, S56 and SX6), which was discussed in a recent publication, also correlates with China-related strains. Fourth, a ^379^STK insertion after position 378 was identified in one of our latest TS1 strains from 2023. Fifth, an insertion occurred after position 1153 (^1154^SLV, associated with a V>P mutation at position 1153, S49 and S51). Finally, single amino acid insertions of asparagine (N) were found after position 135 in most samples (except strain S11, which features a D) and after position 718 in strains S58–59, S61, S62, and S64 (whereas S55 and S60 feature an S). Except for ^1154^SLV, structural predictions placed most insertions on the exposed surface of the spike protein, indicating a potential role in modulating viral immunogenicity. Although the 719N insertion generated an Asn-Xaa-Ser/Thr sequon, its glycosylation potential remains low and could not be predicted by screening software [25]. Except for the common insertions of G2 after positions 55 and 135, all strains containing an insertion were classified as insertion strains in this study Table S4A.

Deletions were observed at 10 sites when compared with the G1 reference sequence (CV777). These included positions 304–305 (in strain S45), 339–349 (in S52, with a partial pattern observed in S44 encompassing positions 340–343, 346, and 348), 376–377 (in S47), 609–612 (in S35, S52, and SX2–4), 669–674 (in S52), 998–1005 (in S52), 1142 (in SX6), and 1193 (in S15, S44–45, S47–52, S55–57, S59–61, and SX5–6). The remaining two sites are considered interchangeable between G2 ^56–58^GVN and G1 ^155–156^DG, where deletions are typically found in other genogroups. However, in this study, distinct deletion characteristics were observed at both sites in strains S34, S41, and SX2-SX4. Furthermore, strains S57 and S58 exhibited a deletion of ^57–58^VN without the presence of ^155–156^DG; thus, we classified these as deletion strains.

**Figure 3:**
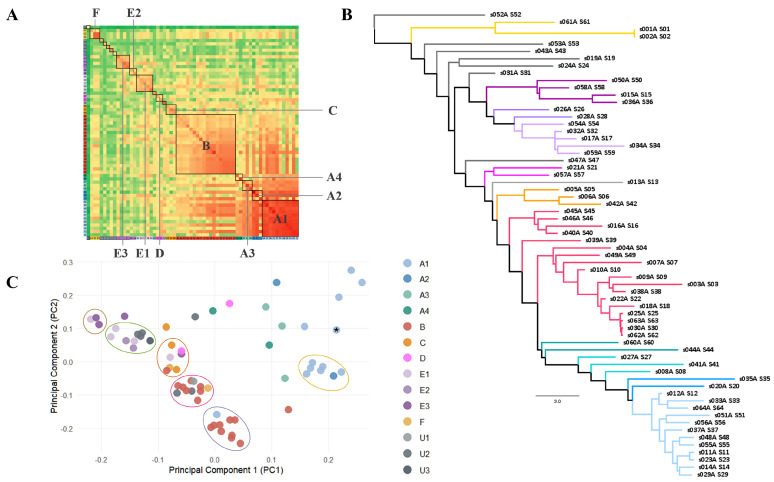
(**A**) Heatmap of the Z-score similarity matrix with clustered classifications based on spike protein structure; the color gradient indicates structural similarity, ranging from green (lowest similarity/low Z-score) to red (highest similarity/high Z-score), with yellow representing the 50th percentile (median similarity). Major structural groups (A1–F) are outlined in black boxes. (**B**) Pseudophylogenetic tree of spike protein structures, with branch colors corresponding to the structural groups (A1–F, U1–U3) indicated in the color legend. (**C**) Multidimensional eigenvector plot (correspondence analysis) of protein clusters analyzed using DALI, color-coded by structural group. Clusters determined by DBSCAN at epsilon = 2.5 are indicated by circles. An asterisk denotes the most predominant group (*n* = 43), for which S55 serves as the representative structure.

Classification in this study was based on a cutoff set at the lowest DALI Z-score value observed against itself, specifically U3 with a Z-score of 42.7 (Figure 3A). Based on this threshold, the spike protein structures identified in Thailand were categorized into 14 subgroups: A (subgroups A1 through A4), B (a variant cluster), C, D, E (subgroups E1 through E3), F, and U (unclassified groups U1 through U3). This classification was supported by the pseudophylogenetic tree generated from DALI, which positioned U1 through U3 as an outgroup (Figure 3B).

Additionally, a scatter plot of multidimensional correspondence vectors was analyzed using the DBSCAN clustering method with an eps value of 2.5 and a minimum sample size of 2, which revealed six distinct structural clusters. Cluster 1 included S01, S02, S05, S06, S16, S50, S57, and S59, while Cluster 2 included S04, S09, S12, S18, S22, S25, S30, S38, S62, and S63. Cluster 3 was composed of S07, S10, S13, S39, S40, S43, S45, S46, S47, S49, and S61. Cluster 4 included S17, S24, S26, S28, S31, S32, S52, S53, and S54, whereas Cluster 5 included S23, S29, S33, S35, S37, S51, and S56. Finally, Cluster 6 comprised S34, S36, and S58, as illustrated in Figure 3C. All clustering and structural protein classification information for these representative sequences is available in Table S2.

**Figure 4:**
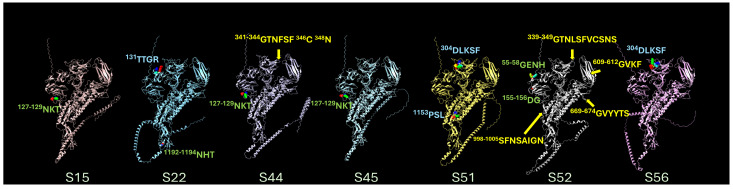
AlphaFold3 structural refinement and characterization of surface loop variations among TH4 InDel variants. Yellow text and arrows indicate deletion sites, blue text indicates insertion sites, and green text highlights amino acid patterns of interest among TH4 strains.

Further comparison of the structural variations among the InDels within the TH4 lineage revealed that strain S52 displays a highly complex epitope profile. It exhibits multiple distinct clusters scattered across the tertiary structure, featuring the unique ^55–58^GENH and ^155–156^DG patterns that resemble those of G1. Concurrently, S52 possesses major deletions, including ^339–349^GTNLSFVCSNS, ^609–612^GVKF, ^669–674^GVYYTS, and a large stalk fragment (^998–1005^SFNSAIGN), making it the shortest spike protein found in Thailand (1353 aa). While strain S44 shares similar deletion regions with S52, it presents a distinct multi-site deletion pattern (^341–344^GTNFSF, ^346^C, and ^348^N). Additionally, a mutation at residues ^127–129^NKT in S15, S44, and S45 introduces a functional N-glycosylation site when compared with the ^127–129^IKT motif found in other strains. Most insertion sites identified within the TH4 InDels are highly accessible on the outer surface of the structure, with the exception of ^1153^PSL (corresponding to ^1153+1^SLV in Supplementary Table S4A) (Figure 4).

Representative tertiary structures of the Porcine Epidemic Diarrhea Virus (PEDV) spike protein monomers (S15, S22, S44, S45, S51, S52, and S56) were modeled using AlphaFold3 to map structural variations. The core protein backbones are displayed as structural ribbons to visualize secondary folds. Green highlights structural motifs that differ from the common G2b (TH4) lineage, specifically pointing out structural variations that impact protein folding or alter potential N-glycosylation sites. Blue indicates insertion sites within the structure, while yellow denotes the positions of deletions.

### 3.3. Chronogram of PEDV Spike Protein Inferred Using BEAST

**Figure 5:**
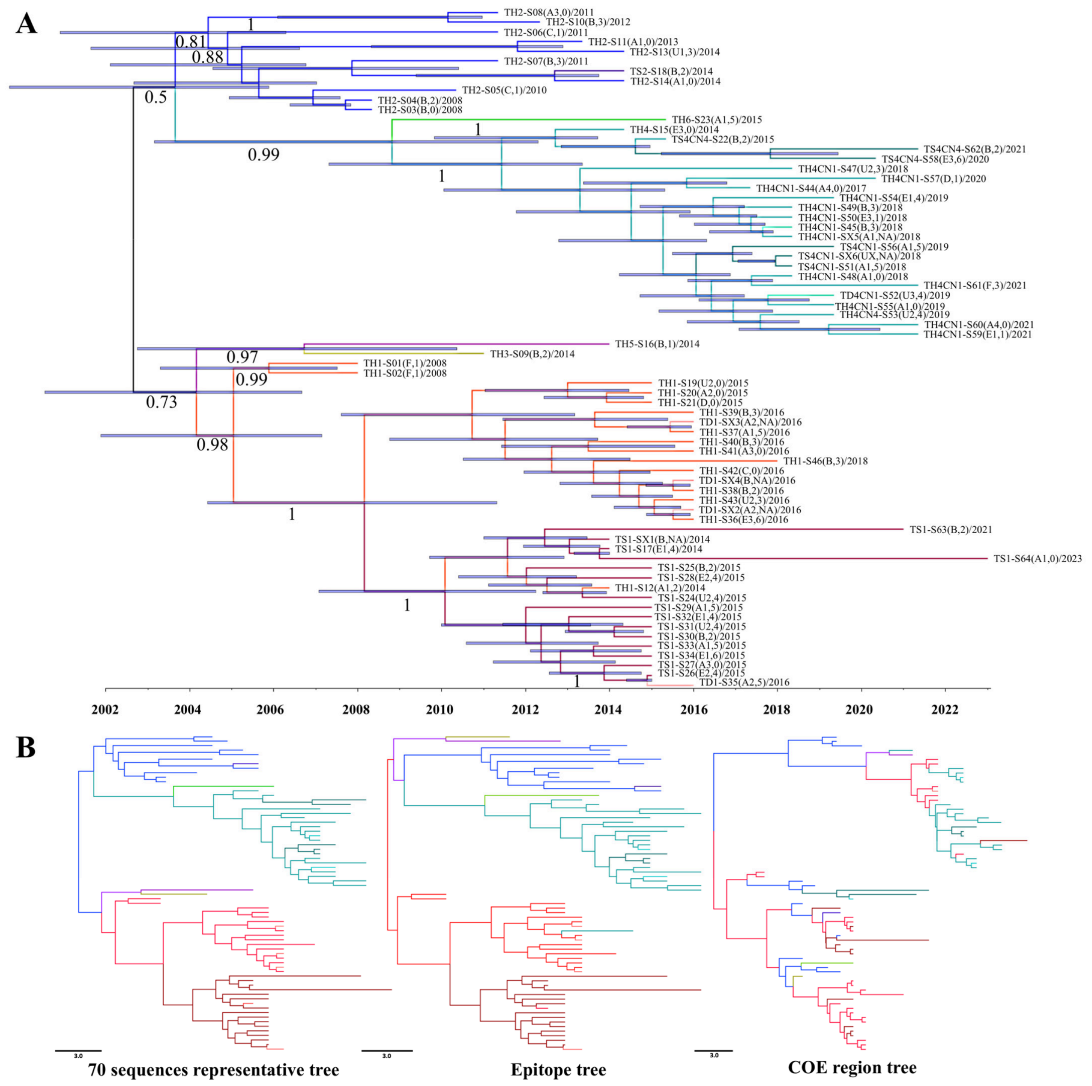
(**A**) Chronogram of the representative tree constructed from 70 sequences using BEAST, showing 95% highest posterior density (HPD) intervals. Tips are labeled with subgroup-structure IDs and year of collection as determined in this study. Brackets indicate structure classification and clustering obtained from DALI. (**B**) Representative phylogenetic tree of the 70 sequences compared alongside trees reconstructed from the epitope and COE regions. The scale bar denotes a period of 3 years. Branch colors indicate Thai lineages: TH1 (red), TH2 (blue), TH3 (gold), TH4 (teal), TH5 (purple), and TH6 (green). Darker or paler shades represent insertions or deletions, respectively. Labels under the branches indicate posterior probability values.

The chronogram obtained from the BEAST analysis showed results consistent with previous nucleotide-based studies. However, the amino acid sequences of the TH1 and TH2 lineages exhibited a divergent evolutionary pattern, and TH2 appeared to be more closely related to TH4, as illustrated in Figure 5A. The chronogram derived from the selected epitope partitions, which included the COE region, closely resembled the complete spike protein chronogram. This indicates that evolutionary signals within these antigenic regions likely reflect the overall evolution of the protein. In contrast, the chronogram based solely on the COE region was inconclusive and did not clearly correspond to the other phylogenetic trees (Figure 5B). The mean substitution rate of the PEDV spike protein amino acids was estimated at 4.102 × 10^−3^ substitutions per site per year. The estimated rates for the epitope and COE regions were 3.829 × 10^−3^ and 2.679 × 10^−3^, respectively.

### 3.4. Predicted Epitope Profiling and Structural Mapping

The epitopes predicted by DiscoTope 3 and ElliPro were aligned with the representative proteins using co-positioning based on S16, a G1 strain with the same length as CV777. Insertion sites were indicated as the previous position +n. The positions of known epitopes used in this study, including COE, SS2, SS6, mAbA3, and mAbG3, were marked, while 2C10 remained outside the detected range. Amino acids were color-coded according to their properties: aliphatic or hydrophobic (A, I, L, M, V), aromatic (F, W, Y), conformationally special (G, P), cysteine (C), hydrophilic (N, Q, S, T), negatively charged (D, E), and positively charged (R, H, K).

**Figure 6:**
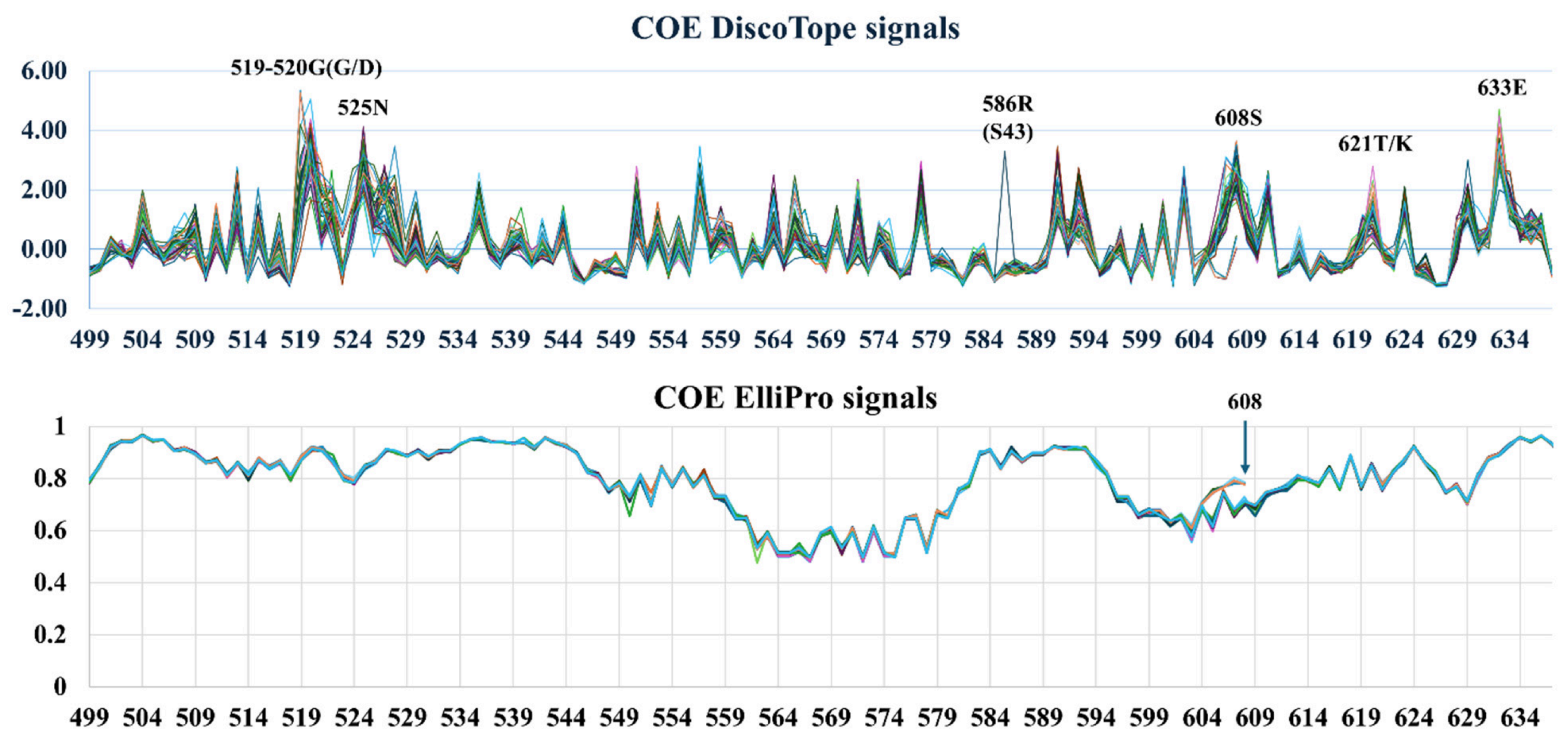
COE epitope signals determined by DiscoTope 3 (**top**) and ElliPro (**bottom**). The *Y*-axis represents the epitope prediction scores, while the *X*-axis indicates the approximate residue positions.

DiscoTope 3 prediction scores were visualized using specific color ranges: 0.4 to 0.9 (orange), 0.9 to 1.5 (dark orange), and greater than 1.5 (red). ElliPro scores were displayed using a gradient from white (0.5) to red (1.0), with orange (0.8) representing the midpoint (Table S4A). The COE region exhibited the highest epitope signals predicted by both tools at residues ^519–528^G[G/D][H/Y/R][S/R][G/S]ANL[I/V/T]A and ^632–637^LEGVTD, with the highest DiscoTope 3 scores observed at sites ^519–520^G[G/D], ^525^N, and ^633^E. Interestingly, the mutation in S43 from ^586^C to ^586^R increased the residue score. Other mutations, such as ^621^T or ^621^K, altered the predicted scores but likely required accompanying structural changes to exert a significant effect. Although several sites predicted by DiscoTope 3 and ElliPro appeared to be correlated, the deletion of residues ^609–612^GVKF typically resulted in a lower DiscoTope signal at ^608^S while slightly increasing the ElliPro score at the same site (Figure 6 and Table S4A).

The discontinuous epitope (DE) alignment revealed differences in some Thai PEDV spike protein structures compared with previous reports. While DE8 was previously described as an eight-residue epitope, in this study, it was calculated as a shorter four-amino acid fragment (^307^DLKS). Additionally, two new epitopes, DE9 and DE10, were included. DE9, which includes ^917–920^SNGR in the reference sequence, was found to be conserved and was consistently predicted as an epitope. DE10 was identified in both InDel and G1 strains found in Thailand at positions ^127–134^IKTLGPTA, adjacent to ^135^N and near the ^155–156^DG deletion. This region was also consistently predicted as an epitope across all samples (Table 1 and Table S4A,B).

Discontinuous epitope analysis suggested that the 70 structures shared 21 distinct pattern types (Table 2 and Supplementary Table S4B). All structures contained DE1 through DE7, although some predicted patterns appeared to be joined (such as DE3 with DE6, or DE4 with DE5), while others appeared fragmented (such as DE6 being divided into DE6a and DE6b). These variations were likely driven by amino acid sequence mutations or structural differences that altered epitope prediction. When predicted epitopes were extended or shortened relative to their baseline patterns, the prediction signal was often weaker than that of the typical pattern. In addition to the conserved DE1 through DE7 core, certain structures featured additional epitopes, specifically DE8, DE9, or DE10, while others did not (Supplementary Table S4B). Strain S56 served as the representative candidate for this DE1–9 profile, with its surface-exposed residues visualized on the predicted structure using UCSF ChimeraX (Figure S2).

Although the predicted epitope patterns provide useful insights into potential antigenic variation among Thai PEDV strains, these computational predictions should be interpreted with caution. Experimental validation, such as monoclonal antibody binding assays, virus neutralization assays, or peptide-based immunological studies, will be required to confirm whether these predicted epitope differences influence antigenicity or immune recognition.

## 4. Discussion

The amino acid-based phylogenetic classification largely aligned with previous nucleotide-based studies regarding the proposed shifts from G2a to G2b [8]. The G1b-InDel was classified into the G1 genogroup although some previous studies classified into G2 genogroup. This discrepancy may result from differences in tree-rooting strategy (e.g., midpoint rooting) or the phylogenetic method that employed, which tends to place the InDel group more closely related to G1a, especially when several novel strains discovered lately tend to be more similar to G1a, for example, G2b-CN4 and G1b-CN2.

The main difference between the S-deletion variant and the common TH1 strains lies in the presence of residues ^55–58^SENH, instead of the ^55–58^[I/T/V]GEN[Q/H] motif, together with the ^155–156^DG motif, which is present in strains S03, S04, and S09. In contrast, some InDel strains (e.g., S35, S41, SX2, SX3, and SX4) lack the ^155–156^DG motif altogether while retaining the G2-associated deletion at this site [8] and the ^55–58^SENH motif. These features suggest that the characteristics of S-InDel strains in Thailand resemble those found in the US and Europe [30]. Interestingly, our findings suggest that the TH2 primitive strains (S03 and S04) may represent an intermediate stage in the transition from G1 to G2, possibly emerging during the early phase of G2a evolution. These strains, detected in 2008, share some features with G1, such as the ^55–58^SENH motif resembling G1’s ^55–58^SMNS, and lack both the insertion at position 55 and the ^155–156^DG deletion. However, their overall genome sequences are more similar to G2, indicating that they may share a common ancestor with the globally observed InDel variants.

Additionally, some other variants at this site include ^55–58^SGENH (S52) and ^55–58^VGENQG (S57 and S58). Interestingly, both S57 and S58, which contain the ^55–58^VGENQG motif, also carry the TTGR insertion between residues 230 and 231, coinciding with the previously described ^231^(N>T) REY insertion, which was linked to a large-scale outbreak in Thailand. S62 also carries this TTGR insertion; however, its flanking motif, ^55–58^IGENQVN, instead resembles that commonly found among the REY-insertion strains, rather than the ^55–58^VGENQG motif seen in S57 and S58. The ^130^(N>T) REY insertion was observed in several strains, including S17–S18, S24–S34, S63–S64, and SX1, and has not been reported in strains outside Thailand. A novel insertion at residue ^303^(V>E) DLKSF, was found in strains S51, S56, and SX6. Additionally, some InDel strains, such as S35, S52, and SX2-SX4 exhibited a deletion of residues ^609–612^GVKF.

We further investigated the recently reported novel epitopes, including mAbA3 and mAbG3 [13,14]. In the mAbA3 region, epitopes were predicted at ^249^[E/D] and ^254^N in all of the representative sequences. The M3S1-0 site, located at position ^183^K, showed a strong and conserved epitope signal across all representative sequences within the same region. Meanwhile, position ^181^Y was predicted to be an epitope site in most representative sequences, whereas position ^182^F was predicted to contribute to an epitope in only some representative sequences. In contrast, the M4S1-BCD site displayed predicted epitope activity only at position ^645^V in fewer than a quarter of representative sequences. Interestingly, position ^655^K, which lies adjacent to the M4S1-BCD region, showed epitope potential in all representative sequences except SX2 and SX3.

Analysis of all 315 Thai PEDV sequences confirmed that the key regions associated with mAbA3 and mAbG3 were largely conserved. The mAbA3 epitope showed the consensus sequence EGFSFNNWFLLS at positions 249–260, with minor variation at positions 249 (E: 311/315; D: 4/315) and 250 (G: 313/315; C: 2/315). The M3S1-0 region showed the consensus sequence KIYHFYFKNDWS at positions 176–187, with variation at positions 179 (H: 280/315; Y: 35/315), 182 (F: 312/315; L: 2/315; S: 1/315), 184 (N: 312/315; T: 3/315), and 185 (D: 312/315; G: 3/315). The M4S1-BCD region showed the consensus sequence LDVCTKYTIYGF at positions 643–654, with variation at positions 644 (D: 309/315; N: 5/315; E: 1/315), 646 (C: 303/315; H: 12/315), and 650 (T: 314/315; I: 1/315). Overall, these findings suggest that the mAbA3- and mAbG3-associated regions are relatively conserved among PEDV strains circulating in Thailand, although limited amino acid variation remains detectable, particularly within the M3S1-0 and M4S1-BCD regions.

The epitope prediction suggests that nearly all sites within the COE region show signals in ElliPro; however, it is challenging to distinguish which positions exhibit stronger signals when compared with the calibrated scores obtained from DiscoTope 3. In this report, we have refined the discontinuous epitope patterns predicted by ElliPro, making them more concise than typical output values and consistent with our previous study on the exotic strain. Some results differ from those of earlier studies, which may be attributed to updates in software tools such as SWISS-MODEL or ElliPro. Nevertheless, the discontinuous epitope (DE) sites identified in previous work remain consistent, and we have compared all structures to identify sites that are consistently observable in each structural model.

In contrast, for the common G2 PEDV, DE10 (^127–134^[I/S/N]KTLGPTA) is located just before the insertion site at ^135^N. All structures containing DE10 were either InDel strains (S09, S35, S41, SX2, and SX3) or the G1 strain (S16), and none of them contained the insertion at site ^135^N. Regarding DE8, although its predicted length is shorter than previously reported, it still exhibits a very strong signal in DiscoTope 3, particularly in exotic strains with the insertions (S51, S56, and SX6), supporting our original hypothesis. While DE1–7 are conserved across all structures, DE8–10 are found only in specific structures. Nevertheless, their epitope signals are consistently predicted by either DiscoTope 3 or ElliPro, except for DE8, which is detected only in structures containing the insertion at site 303.

Based on structural protein classification, the primitive strains found in Thailand were classified into group B (G2a-TH2) and group F (G2a-TH1). Although group B strains were clustered together, the sequences within this group showed high variation and tend to separate into at least two subgroups. Some members of group B may even resemble the protein structure of other groups, particularly group A. The DALI results (Figure 3 and Figure 5, Tables S2 and S4A) varied and did not correlate well with those obtained using other classification methods. This discrepancy may be due to DALI’s focus on evaluating backbone atoms, particularly the C-alpha coordinates [31]. In our study, the protein structures were modeled using SWISS-MODEL based on homology-modeling templates. These models are likely to have conserved backbones [22], though we compared them with other methods recommended by the DALI developers. We found that these alternative similarity scores were higher than those obtained via SWISS-MODEL. Standard homology modeling can sometimes mask subtle structural shifts by forcing sequences to fit a known template. To overcome this limitation, using advanced modeling or new generative AI could provide the higher discriminatory power needed to detect genuine structural differences. These methods are better at predicting realistic structural variations, so future research should apply them in a smaller, more focused group of sequences to clearly see how specific side-chain changes affect the virus.

Furthermore, we found that many structural types did not correlate well with amino acid sequence similarity. For example, S19 (U2), S20 (A2), and S21 (D), which belong to the first monophyletic group within the TH1 cluster, exhibited highly similar structures despite sequence differences. However, structural variation was mainly associated with changes in residue properties. Notably, comparisons between S19 (U2) and S20 (A2) revealed important substitutions, Y699F and T776I, that altered the physicochemical properties of these residues. Additionally, when comparing the structure of S20 (A2) with that of the deletion strain S35 (A2), which shares the same overall structure type, we observed that structural differences were primarily localized to the deletion region, while the rest of the structure remained unchanged (Figure S1). To expand on these findings, incorporating molecular dynamics (MD) simulations in future studies will be a vital step in evaluating how the conformational flexibility of these specific side-chain variations directly impacts epitope accessibility.

Notably, representative sequence S55 warrants separate discussion. Chosen to represent the largest group comprising 43 samples, S55 was initially classified as an outlier by DBSCAN. However, PCA revealed that this structure lies very close to Cluster 5, which defines the predominant A1 subtype. Given that S55 is also classified as an A1 subtype, these findings strongly indicate that Cluster 5 and the A1 subtype represent the predominant structural group within studied population. This conclusion is further supported by our structural mapping: the most prevalent DE pattern (Table 2) shares an identical conformation with S55.

From our perspective, mapping the structural trajectories of circulating field variants provides essential baseline context for vaccine design and regional PEDV control. In this study, Cluster 5 emerges as the predominant structural framework, as it demonstrates a consistent structural correlation with a diverse array of strains, spanning the TH1, TH2, TH4, and TH6 lineages, isolated from past outbreaks up to the present day. While variations trending toward other structural groups, such as the epidemic-associated Clusters 2 and 3, or the post-pandemic, China-like signatures in Clusters 4 and 6, suggest ongoing structural adaptation, the discriminatory power of our current in silico profiling pipeline cannot definitively resolve these subtler transitions. Consequently, speculative conclusions regarding these specific cluster shifts are deliberately avoided. Nevertheless, the persistent prevalence of the Cluster 5 architecture closely correlates with the most highly diverse variant group of PEDV in Thailand, highlighting the necessity of selecting immunogens that structurally match this enduring surface landscape.

Nevertheless, since we applied a stringent threshold, structural classification was still possible. For instance, primitive TH1 (S01 and S02), classical TH2 (S05 and S06), and G1a-TH5 (S16) were clustered together by DBSCAN and assigned to subtypes F, C, and B, respectively. Although the primitive TH2 strain (S04) was classified in Cluster 2, it remains close to Cluster 1. In contrast, S03 was considered noise but was considered as subtype B. These results suggest that protein structure evolution may have progressed from Cluster 1 to other clusters.

Because the present analyses relied exclusively on computational structural prediction, the identified discontinuous epitopes should be considered putative until experimentally validated. Future studies incorporating antibody binding assays, virus neutralization assays, and more advanced structural modeling approaches will be necessary to determine whether the predicted structural differences alter antibody recognition and to better resolve the associated structural changes.

## 5. Conclusions

In conclusion, this study provides an updated amino acid-based, structural, and epitope-focused analysis of PEDV spike proteins circulating in Thailand. By integrating newly obtained outbreak sequences with previously reported Thai PEDV data, we identified additional spike variations, including deletion- and InDel-related patterns, and confirmed that several findings were consistent with earlier molecular epidemiological studies. Comparative analysis also suggested that the primitive TH2 strains share features with G1 or US-InDel-like strains, although they remain classified within the G2 lineage and show relationships with G2b-associated strains.

Predicted structural classification indicated that primitive TH1, classical TH2, and G1a-TH5 strains shared relatively high structural similarity. These findings suggest that Cluster 1 may represent an early or ancestral-like structural pattern among PEDV strains circulating in Thailand, although this interpretation should be confirmed using more rigorous structural approaches and direct experimental validation.

To translate these findings into field applications, the widespread presence of Cluster 5 strains makes this group the most important target for future vaccine strain selection. Furthermore, because minor changes in these structural clusters and surrounding epitope areas can influence vaccine effectiveness, these results show that veterinarians should screen viral sequences before using live-virus feedback protocols, rather than relying on empirical decision-making.

Overall, this updated analysis provides a structural and bioinformatic framework for monitoring PEDV spike evolution in Thailand. However, a limitation of this study is the lack of direct correlation analysis with in vitro or in vivo phenotypic data. Therefore, these computational findings should be regarded as a preliminary guide, and further experimental studies, including antibody binding and virus neutralization assays, remain essential to validate the predicted epitopes and determine how specific spike protein changes affect viral virulence, antigenicity, and host immune responses.

## Figures and Tables

**Table 1:** Structural features of discontinuous epitope (DE) patterns within representative sequences, including total residues.

DE	Found in	Min	Avg	Max
1	66	165	168	171
1 + 3	1	199		
1 + 3 + 6	1	225		
1 + 7	2	181	182	182
2	70	185	194	195
3	47	31	31	32
3 + 6	14	54	55	56
3 + 6a	7	50	51	51
4	54	75	79	85
4 + 5	16	193	194	196
5	54	112	116	120
6	25	22	22	23
6a	23	16	18	18
6b	30	5	5	6
7	68	12	12	14
8	3	4	4	4
9	40	4	4	5
10	6	8	8	8

**Table 2:** Distribution of discontinuous epitope patterns, sample counts, and structural IDs across the analyzed representative sequences.

Pattern	Discontinuous Epitopes	Samples	Structure ID
1	DE1, DE2, DE3, DE4, DE5, DE6a, DE6b, DE7, DE9	12	S38, S40, S44, S45, S47, S48, S50, S55, S57, S60, SX4, SX5
2	DE1, DE2, DE3, DE4, DE5, DE6, DE7	12	S12, S19, S21, S23, S36, S37, S39, S42, S46, S53, S54, S58
3	DE1, DE2, DE3, DE4, DE5, DE6, DE7, DE9	9	S05, S06, S07, S08, S10, S11, S13, S14, S20
4	DE1, DE2, DE3 + DE6, DE4 + DE5, DE7	8	S17, S25, S26, S31, S32, S33, S63, SX1
5	DE1, DE2, DE3 + DE6a, DE4, DE5, DE6b, DE7, DE9	5	S03, S04, S43, S52, S61
6	DE1, DE2, DE3, DE4, DE5, DE6a, DE6b, DE7, DE8, DE9	3	S51, S56, SX6
7	DE1, DE10, DE2, DE3, DE4, DE5, DE6a, DE6b, DE7, DE9	3	S35, SX2, SX3
8	DE1, DE2, DE3 + DE6, DE4, DE5, DE7	2	S22, S62
9	DE1, DE2, DE3, DE4 + DE5, DE6a, DE6b, DE7	2	S27, S28
10	DE1, DE2, DE3 + DE6, DE4, DE5, DE7, DE9	2	S01, S02
11	DE1, DE10, DE2, DE3 + DE6a, DE4, DE5, DE6b, DE7, DE9	2	S16, S41
12	DE1 + DE3, DE2, DE4, DE5, DE6, DE7	1	S15
13	DE1, DE2, DE3 + DE6, DE4 + DE5, DE7, DE9	1	S30
14	DE1, DE2, DE3, DE4 + DE5, DE6, DE7	1	S24
15	DE1, DE2, DE3, DE4 + DE5, DE6, DE7, DE9	1	S18
16	DE1, DE2, DE3, DE4 + DE5, DE6a, DE6b, DE7, DE9	1	S29
17	DE1, DE10, DE2, DE3 + DE6, DE4, DE5, DE7, DE9	1	S09
18	DE1 + DE7, DE2, DE3, DE4, DE5, DE6a, DE6b	1	S59
19	DE1, DE2, DE3, DE4, DE5, DE6a, DE6b, DE7	1	S49
20	DE1 + DE7, DE2, DE3, DE4 + DE5, DE6	1	S64
21	DE1 + DE3 + DE6, DE2, DE4 + DE5, DE7	1	S34

## Data Availability

All primary data supporting the conclusions of this study are included within the article and its additional Supplementary Files. Further underlying datasets or specific materials that support the findings of this research are available from the corresponding author on reasonable request.

## Supplementary Materials

The following supporting information can be downloaded at https://www.mdpi.com/article/10.3390/ani16152314/s1, Figure S1 shows protein structures determined using iCn3D, based on the normalized hydrophobicity of selected structures. The results demonstrate that even a few amino acid changes can affect the predicted subtype, as seen in S19 (TH1) and S20 (TH1). Additionally, structural changes were observed due to a deletion in the pandemic strain from 2015, with TS1-S26 (TS1) evolving into S35 (TD1). Figure S2 shows the discontinuous epitopes (DE) predicted using ElliPro in this study. The main panels demonstrate the structure of S56 (MW805354), revealing DE1-9. The inset panels compare a local structural variation: the lower small panel highlights DE10 (teal) present in the G2a lineage strain S09 (KX981899), demonstrating that this site tends to project outward when compared with the corresponding loop structure of S56 shown in the upper small panel. Figure S3 Heatmap of the Z-score similarity matrix with clustered classifications based on spike protein structure. Table S1 summary of the samples used in this study. Subsections include: (A) classification of global sequences by group and year; (B) classification of Thai sequences by group and year; (C) summary of sample sequences used in this study; and (D) classification of sample sequences within each subgroup. Table S2 provides a list of the sequence and structure ID, in the ‘Population’ column showed the sequence in this study those grouping by the CD-HIT clusters, red indicates clusters that include sequences with length variations, while blue indicates additional sequences not included in the CD-HIT clustering results at 98% identity. ‘NA’ denotes information not available for SX structures, as they were analyzed separately. The protein subtype and clusters showed the result demonstrated by the pseudophylogenetic tree and Multidimensional eigenvector plot obtained by DALI, respectively. Table S3 spatial distribution and provincial dominance matrix of clustered representative lineages. Clusters are stratified by Structure ID and mapped to their corresponding master Sequence ID. Geographical distribution is tiered into Primary, Secondary, and Tertiary columns based on the sample frequency within each individual sequence cluster. Table S4 (A) amino acid sequences aligned with the G1 reference group. Epitope positions investigated in this study are indicated, and insertion sites found in Thai PEDV strains are marked as position +n. Deletion sites are labeled in blue with red text. Amino acids are color-coded according to their properties, as explained in the legend at the bottom of the table. Column headers indicate the structure IDs of the 70 protein sequences used in this study. The sub-columns R, D, and E represent the residue, DiscoTope signal (cutoff 0.6), and ElliPro signal (cutoff 0.5), respectively. (B) Refined DE patterns identified in this study, including DE region information.

## References

[1] Pensaert M.B., de Bouck P. (1978). A new coronavirus-like particle associated with diarrhea in swine. Arch. Virol..

[2] Chasey D., Cartwright S.F. (1978). Virus-like particles associated with porcine epidemic diarrhoea. Res. Vet. Sci..

[3] Pospischil A., Stuedli A., Kiupel M. (2002). Update on porcine epidemic diarrhea. J. Swine Health Prod..

[4] Luo H., Liang Z., Lin J., Wang Y., Liu Y., Mei K., Zhao M., Huang S. (2024). Research progress of porcine epidemic diarrhea virus S protein. Front. Microbiol..

[5] Stevenson G.W., Hoang H., Schwartz K.J., Burrough E.R., Sun D., Madson D., Cooper V.L., Pillatzki A., Gauger P., Schmitt B.J. (2013). Emergence of Porcine epidemic diarrhea virus in the United States: Clinical signs, lesions, and viral genomic sequences. J. Vet. Diagn. Investig..

[6] Kocherhans R., Bridgen A., Ackermann M., Tobler K. (2001). Completion of the porcine epidemic diarrhoea coronavirus (PEDV) genome sequence. Virus Genes.

[7] Sun M., Ma J., Wang Y., Wang M., Song W., Zhang W., Lu C., Yao H. (2015). Genomic and epidemiological characteristics provide new insights into the phylogeographical and spatiotemporal spread of porcine epidemic diarrhea virus in Asia. J. Clin. Microbiol..

[8] Lee C. (2015). Porcine epidemic diarrhea virus: An emerging and re-emerging epizootic swine virus. Virol. J..

[9] Stott C.J., Temeeyasen G., Tripipat T., Kaewprommal P., Tantituvanont A., Piriyapongsa J., Nilubol D. (2017). Evolutionary and epidemiological analyses based on spike genes of porcine epidemic diarrhea virus circulating in Thailand in 2008–2015. Infect. Genet. Evol..

[10] Yu L., Liu Y., Wang S., Zhang L., Liang P., Wang L., Dong J., Song C. (2020). Molecular Characteristics and Pathogenicity of Porcine Epidemic Diarrhea Virus Isolated in Some Areas of China in 2015–2018. Front. Vet. Sci..

[11] Chang S.H., Bae J.L., Kang T.J., Kim J., Chung G.H., Lim C.W., Laude H., Yang M.S., Jang Y.S. (2002). Identification of the epitope region capable of inducing neutralizing antibodies against the porcine epidemic diarrhea virus. Mol. Cells.

[12] Sun D., Feng L., Shi H., Chen J., Cui X., Chen H., Liu S., Tong Y., Wang Y., Tong G. (2008). Identification of two novel B cell epitopes on porcine epidemic diarrhea virus spike protein. Vet. Microbiol..

[13] Thavorasak T., Chulanetra M., Glab-Ampai K., Teeranitayatarn K., Songserm T., Yodsheewan R., Sae-Lim N., Lekcharoensuk P., Sookrung N., Chaicumpa W. (2022). Novel Neutralizing Epitope of PEDV S1 Protein Identified by IgM Monoclonal Antibody. Viruses.

[14] Thavorasak T., Chulanetra M., Glab-Ampai K., Mahasongkram K., Sae-Lim N., Teeranitayatarn K., Songserm T., Yodsheewan R., Nilubol D., Chaicumpa W. (2022). Enhancing epitope of PEDV spike protein. Front. Microbiol..

[15] Kirchdoerfer R.N., Bhandari M., Martini O., Sewall L.M., Bangaru S., Yoon K.J., Ward A.B. (2021). Structure and immune recognition of the porcine epidemic diarrhea virus spike protein. Structure.

[16] Ma Z., Li Z., Li Y., Zhao X., Zheng C., Li Y., Guo X., Xu L., Zheng Z., Liu G. (2025). Changes in the motifs in the D0 and SD2 domains of the S protein drive the evolution of virulence in enteric coronavirus porcine epidemic diarrhea virus. J. Virol..

[17] Cheun-Arom T., Temeeyasen G., Tripipat T., Kaewprommal P., Piriyapongsa J., Sukrong S., Chongcharoen W., Tantituvanont A., Nilubol D. (2016). Full-length genome analysis of two genetically distinct variants of porcine epidemic diarrhea virus in Thailand. Infect. Genet. Evol..

[18] Holm L., Laiho A., Toronen P., Salgado M. (2023). DALI shines a light on remote homologs: One hundred discoveries. Protein Sci..

[19] Fu L., Niu B., Zhu Z., Wu S., Li W. (2012). CD-HIT: Accelerated for clustering the next-generation sequencing data. Bioinformatics.

[20] Price M.N., Dehal P.S., Arkin A.P. (2009). FastTree: Computing large minimum evolution trees with profiles instead of a distance matrix. Mol. Biol. Evol..

[21] Suchard M.A., Lemey P., Baele G., Ayres D.L., Drummond A.J., Rambaut A. (2018). Bayesian phylogenetic and phylodynamic data integration using BEAST 1.10. Virus Evol..

[22] Waterhouse A., Bertoni M., Bienert S., Studer G., Tauriello G., Gumienny R., Heer F.T., de Beer T.A.P., Rempfer C., Bordoli L. (2018). SWISS-MODEL: Homology modelling of protein structures and complexes. Nucleic Acids Res..

[23] Ester M., Kriegel H.-P., Sander J., Xu X. A density-based algorithm for discovering clusters in large spatial databases with noise. Proceedings of the Second International Conference on Knowledge Discovery and Data Mining.

[24] Abramson J., Adler J., Dunger J., Evans R., Green T., Pritzel A., Ronneberger O., Willmore L., Ballard A.J., Bambrick J. (2024). Accurate structure prediction of biomolecular interactions with AlphaFold 3. Nature.

[25] Gupta R., Brunak S. (2002). Prediction of glycosylation across the human proteome and the correlation to protein function. Pac. Symp. Biocomput..

[26] Pettersen E.F., Goddard T.D., Huang C.C., Meng E.C., Couch G.S., Croll T.I., Morris J.H., Ferrin T.E. (2021). UCSF ChimeraX: Structure visualization for researchers, educators, and developers. Protein Sci..

[27] Hoie M.H., Gade F.S., Johansen J.M., Wurtzen C., Winther O., Nielsen M., Marcatili P. (2024). DiscoTope-3.0: Improved B-cell epitope prediction using inverse folding latent representations. Front. Immunol..

[28] Ponomarenko J., Bui H.H., Li W., Fusseder N., Bourne P.E., Sette A., Peters B. (2008). ElliPro: A new structure-based tool for the prediction of antibody epitopes. BMC Bioinform..

[29] Stott C.J., Jermsutjarit P., Pornpanom P., Lin H., Tantituvanont A., Nilubol D. (2025). Detection and Phylogenetic Analysis of an Exotic Strain of Porcine Epidemic Diarrhea Virus and Its Effect on an Affected Herd Immunized Against the Endemic Strain in Thailand. Animals.

[30] Wang L., Byrum B., Zhang Y. (2014). New variant of porcine epidemic diarrhea virus, United States, 2014. Emerg. Infect. Dis..

[31] Holm L. (2020). DALI and the persistence of protein shape. Protein Sci..

