# Supplementary material for: Classification Evolution and Epitope Prediction of the Porcine Epidemic Diarrhea Virus (PEDV) Spike Protein in Thailand (2008–2024): Updated Insights for Preventive Strategies
Source: Animals (Basel). 2026 Jul 27;16(15):2314. doi: 10.3390/ani16152314 (PMC13463572; doi:10.3390/ani16152314)
Supplement: Supplementary file 1 [file animals-16-02314-s001.zip › Figure S1 and S2.pdf]

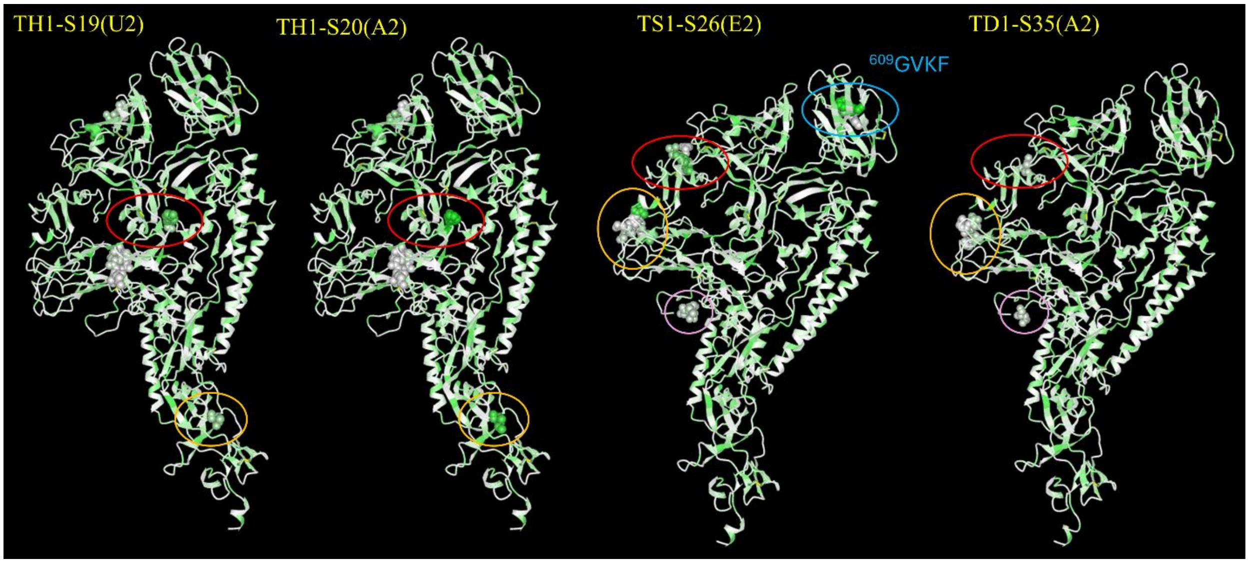

Figure S1 shows protein structures determined using iCn3D, based on the normalized hydrophobicity of selected structures. The results demonstrate that even a few amino acid changes can affect the predicted subtype, as seen in S19 (TH1) and S20 (TH1). Additionally, structural changes were observed due to a deletion in the pandemic strain from 2015, with TS1-S26 (TS1) evolving into S35 (TD1).

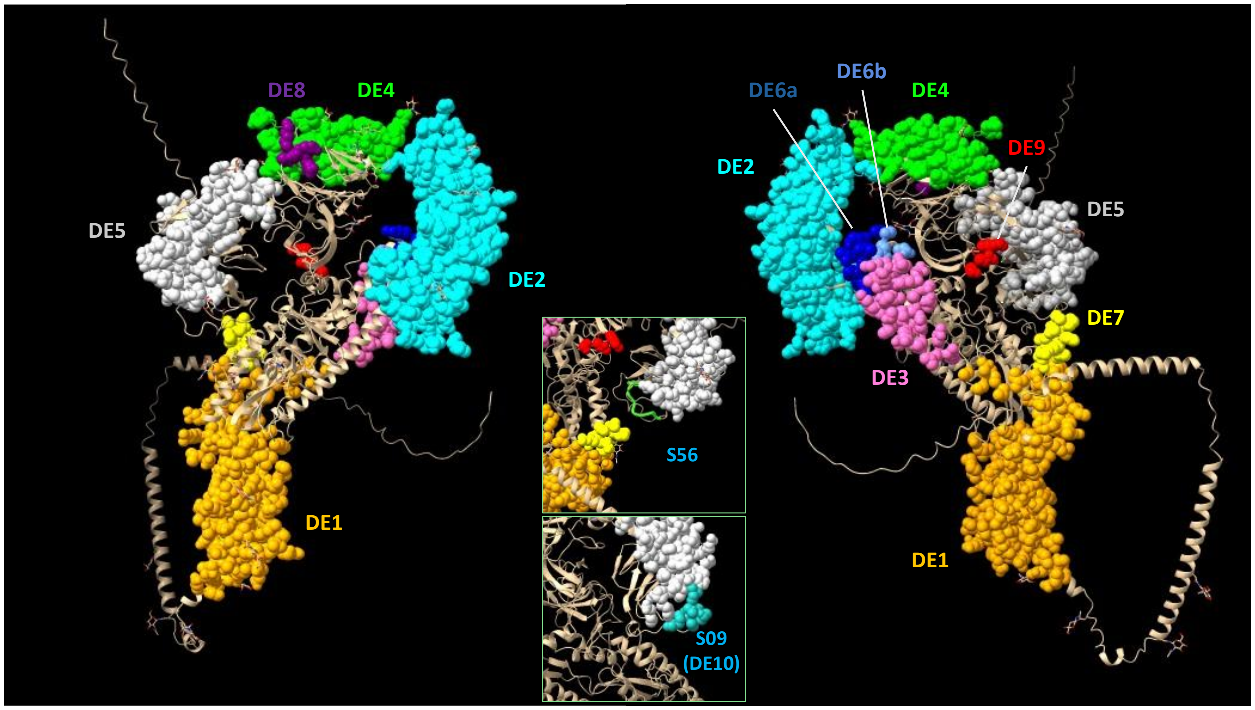

Figure S2 shows the discontinuous epitopes (DE) predicted using ElliPro in this study. The main panels demonstrate the structure of S56 (MW805354), revealing DE1-9. The inset panels compare a local structural variation: the lower small panel highlights DE10 (teal) present in the G2a lineage strain S09 (KX981899), demonstrating that this site tends to project outward when compared to the corresponding loop structure of S56 shown in the upper small panel.
