# Supplementary material for: Classification Evolution and Epitope Prediction of the Porcine Epidemic Diarrhea Virus (PEDV) Spike Protein in Thailand (2008–2024): Updated Insights for Preventive Strategies
Source: Animals (Basel). 2026 Jul 27;16(15):2314. doi: 10.3390/ani16152314 (PMC13463572; doi:10.3390/ani16152314)

Figure S3 Heatmap of the Z-score similarity matrix with clustered classifications based on spike protein structure.

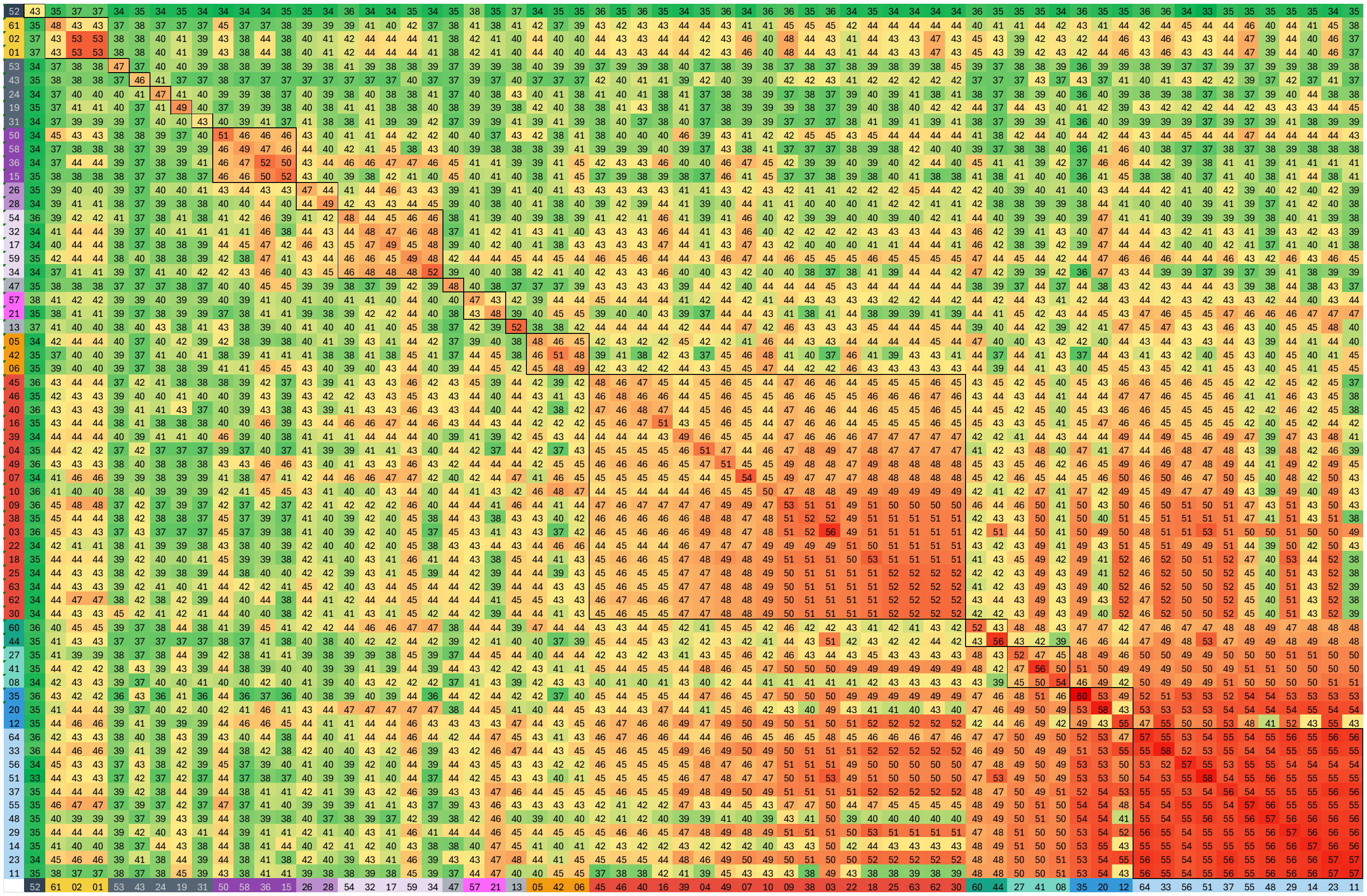

Supplement: Supplementary file 1 [file animals-16-02314-s001.zip › Figure S3 (supplementary material).pdf]
