# Supplementary material for: Classification Evolution and Epitope Prediction of the Porcine Epidemic Diarrhea Virus (PEDV) Spike Protein in Thailand (2008–2024): Updated Insights for Preventive Strategies
Source: Animals (Basel). 2026 Jul 27;16(15):2314. doi: 10.3390/ani16152314 (PMC13463572; doi:10.3390/ani16152314)
Supplement: Supplementary file 1 [file animals-16-02314-s001.zip › Table S2 and S3.pdf]

Table S2 provides a list of the sequence and structure ID, in the 'Population' column showed the sequence in this study those grouping by the CD-HIT clusters, red indicates clusters that include sequences with length variations, while blue indicates additional sequences not included in the CD-HIT clustering results at 98% identity. 'NA' denotes information not available for SX structures, as they were analyzed separately. The protein subtype and clusters showed the result demonstrated by the pseudophylogenetic tree and Multidimensional eigenvector plot obtained by DALI, respectively.

Structure ID	Sequence ID	Population	Protein cluster	Protein subtype	Length
S01	2008/TH1/KC764952	1	1	F	1385
S02	2008/TH1/KC764953	1	1	F	1385
S03	2008/TH2/KY000559	1	N	B	1382
S04	2008/TH2/KY828922	1	2	B	1382
S05	2010/TH2/KC764955	2	1	C	1385
S06	2011/TH2/KC764956	1	1	C	1385
S07	2011/TH2/KC764957	4	3	B	1385
S08	2011/TH2/KF724937	5	N	A3	1385
S09	2011/TH3/KX981899	1	2	B	1382
S10	2012/TH2/KF724936	1	3	B	1385
S11	2013/TH2/121301A	8	N	A1	1385
S12	2014/TH1/041401A	4	2	A1	1385
S13	2014/TH2/011401A	3	3	U1	1384
S14	2014/TH2/091401A	2	N	A1	1385
S15	2014/TD4/KR610993	2	N	E3	1384
S16	2014/TH5/KR610991	2	1	B	1382
S17	2014/TS1/KX981897	26	4	E1	1388
S18	2014/TS2/KX981898	2	2	B	1388
S19	2015/TH1/021503A	6	N	U2	1385
S20	2015/TH1/051501A	3	N	A2	1385
S21	2015/TH1/051502B	1	N	D	1385
S22	2015/TS4CN4/MZ090589	10	2	B	1389
S23	2015/TH6/KX981900	1	5	A1	1385
S24	2015/TS1/011502A	2	4	U2	1388
S25	2015/TS1/011521A	9	2	B	1388
S26	2015/TS1/021501A	**2(-1)**	4	E2	1388
S27	2015/TS1/021601B	12	N	A3	1388
S28	2015/TS1/021502A	4	4	E2	1388
S29	2015/TS1/071501A	4	5	A1	1388
S30	2015/TS1/071501C	2	2	B	1388
S31	2015/TH1/081501B	1	4	U2	1388
S32	2015/TH1/121501C	1	4	E1	1388
S33	2015/TH1/121501B	1	5	A1	1388
S34	2015/TH1/121501A	3	6	E1	1388
S35	2016/TD1/021601S	**0(+1)**	5	A2	1376
S36	2016/TH1/021601M	**6(-1)**	6	E3	1385
S37	2016/TH1/021601D	**6(-1)**	5	A1	1385

Structure ID	Sequence ID	Population	Protein cluster	Protein subtype	Length
S38	2016/TH1/021601L	**6(-1)**	2	B	1385
S39	2016/TH1/021601B	7	3	B	1385
S40	2016/TH1/021604A	6	3	B	1385
S41	2016/TH1/031601A	1	N	A3	1380
S42	2016/TH1/071601A	2	N	C	1385
S43	2016/TH1/111601A	2	3	U2	1385
S44	2017/TH4CN1/011701A	2	N	A4	1378
S45	2018/TH4CN1/071803A	**0(+1)**	3	B	1381
S46	2018/TH1/071802A	21	3	B	1385
S47	2018/TH4CN1/021801A	5	3	U2	1382
S48	2018/TH4CN1/021801B	**0(+1)**	N	A1	1384
S49	2018/TH4CN1/071806A	1	3	B	1384
S50	2018/TH4CN1/071807A	1	1	E3	1384
S51	2018/TS4CN1/071801A	**2(-1)**	5	A1	1392
S52	2019/TD4CN1/041901A	3	4	U3	1353
S53	2019/TH4CN4/091901A	6	4	U2	1385
S54	2019/TH4CN4/091902A	**8(-2)**	4	E1	1385
S55	2019/TH4CN1/041910A	**43(-1)**	N	A1	1385
S56	2019/TS4CN1/MW805354	4	5	A1	1389
S57	2020/TS4CN1/062001A	6	1	D	1386
S58	2020/TS4CN4/012011A	2	6	E3	1388
S59	2021/TH4CN1/012105A	3	1	E1	1385
S60	2021/TH4CN1/012108A	3	N	A4	1385
S61	2021/TH4CN1/012109A	32	3	F	1385
S62	2021/TS4CN4/012110A	2	2	B	1390
S63	2021/TS1/032101A	**5(-1)**	2	B	1388
S64	2023/TS1/012302A	1	N	A1	1392
SX1	2014/TS1/071416A	**0(+1)**	NA	NA	1387
SX2	2016/TD1/021602C	**0(+1)**	NA	NA	1376
SX3	2016/TD1/021601D	**0(+1)**	NA	NA	1376
SX4	2016/TD1/021602L	**0(+1)**	NA	NA	1376
SX5	2018/TH4CN1/071804A	**0(+1)**	NA	NA	1384
SX6	2018/TS4CN1/071808A	**0(+1)**	NA	NA	1391

Table S3 spatial distribution and provincial dominance matrix of clustered representative lineages. Clusters are stratified by Structure ID and mapped to their corresponding master Sequence ID. Geographical distribution is tiered into Primary, Secondary, and Tertiary columns based on the sample frequency within each individual sequence cluster.

Structure ID	Sequence ID	Primary Province (Dominant)	Secondary Province	Tertiary Province
S01	2008/TH1/KC764952	Ratchaburi (1)		
S02	2008/TH1/KC764953	Ratchaburi (1)		
S03	2008/TH2/KY000559	Ratchaburi (1)		
S04	2008/TH2/KY828922	Ratchaburi (1)		
S05	2010/TH2/KC764955	Ratchaburi (2)		
S06	2011/TH2/KC764956	Ratchaburi (1)		
S07	2011/TH2/KC764957	Saraburi (2)<br>Ratchaburi (2)		
S08	2011/TH2/KF724937	Ratchaburi (5)		
S09	2011/TH3/KX981899	Saraburi (1)		
S10	2012/TH2/KF724936	Ratchaburi (1)		
S11	2013/TH2/121301A	Ratchaburi (4)<br>Saraburi (4)		
S12	2014/TH1/041401A	Chonburi (2)	Chachoengsao (1)<br>Nakhon Ratchasima (1)	
S13	2014/TH2/011401A	Saraburi (3)		
S14	2014/TH2/091401A	Saraburi (2)		
S15	2014/TD4/KR610993	Chonburi (2)		
S16	2014/TH5/KR610991	Chonburi (2)		
S17	2014/TS1/KX981897	Ratchaburi (25)	Nakhon Pathom (1)	
S18	2014/TS2/KX981898	Ratchaburi (2)		
S19	2015/TH1/021503A	Saraburi (4)	Buriram (2)	
S20	2015/TH1/051501A	Chanthaburi (3)		
S21	2015/TH1/051502B	Chanthaburi (1)		
S22	2015/TS4CN4/MZ090589	Nakhon Pathom (10)		
S23	2015/TH6/KX981900	Ratchaburi (1)		
S24	2015/TS1/011502A	Buriram (2)		
S25	2015/TS1/011521A	Ratchaburi (6)	Buriram (3)	
S26	2015/TS1/021501A	Saraburi (2)		
S27	2015/TS1/021601B	Saraburi (4)	Chonburi (3)<br>Lopburi (3)	Ratchaburi (1)<br>Nakhon Pathom (1)
S28	2015/TS1/021502A	Ratchaburi (3)	Saraburi (1)	
S29	2015/TS1/071501A	Ratchaburi (4)		
S30	2015/TS1/071501C	Ratchaburi (2)		
S31	2015/TH1/081501B	Saraburi (1)		
S32	2015/TH1/121501C	Chonburi (1)		
S33	2015/TH1/121501B	Saraburi (1)		
S34	2015/TH1/121501A	Lopburi (2)	Ratchaburi (1)	
S35	2016/TD1/021601S	S26		

Structure ID	Sequence ID	Primary Province (Dominant)	Secondary Province	Tertiary Province
S36	2016/TH1/021601M	Chachoengsao (6)		
S37	2016/TH1/021601D	Saraburi (4)	Ratchaburi (2)	
S38	2016/TH1/021601L	Nakhon Ratchasima (6)		
S39	2016/TH1/021601B	Nakhon Panom (7)		
S40	2016/TH1/021604A	Nakhon Panom (6)		
S41	2016/TH1/031601A	Ratchaburi (1)		
S42	2016/TH1/071601A	Ratchaburi (2)		
S43	2016/TH1/111601A	Ratchaburi (2)		
S44	2017/TH4CN1/011701A	Ratchaburi (2)		
S45	2018/TH4CN1/071803A	S54		
S46	2018/TH1/071802A	Ratchaburi (21)		
S47	2018/TH4CN1/021801A	Ratchaburi (5)		
S48	2018/TH4CN1/021801B	S61		
S49	2018/TH4CN1/071806A	Ratchaburi (1)		
S50	2018/TH4CN1/071807A	Ratchaburi (1)		
S51	2018/TS4CN1/071801A	Ratchaburi (2)		
S52	2019/TD4CN1/041901A	Ratchaburi (3)		
S53	2019/TH4CN4/091901A	Ratchaburi (6)		
S54	2019/TH4CN4/091902A	Ratchaburi (8)		
S55	2019/TH4CN1/041910A	Ratchaburi (39)	Saraburi (4)	
S56	2019/TS4CN1/MW805354	Saraburi (4)		
S57	2020/TS4CN1/062001A	Ratchaburi (6)		
S58	2020/TS4CN4/012011A	Ratchaburi (2)		
S59	2021/TH4CN1/012105A	Ratchaburi (3)		
S60	2021/TH4CN1/012108A	Ratchaburi (3)		
S61	2021/TH4CN1/012109A	Ratchaburi (31)	Unknown (1)	
S62	2021/TS4CN4/012110A	Ratchaburi (2)		
S63	2021/TS1/032101A	Ratchaburi (5)		
S64	2023/TS1/012302A	Ratchaburi (1)		
SX1	2014/TS1/071416A	**S63**		
SX2	2016/TD1/021602C	**S36**		
SX3	2016/TD1/021601D	**S37**		
SX4	2016/TD1/021602L	**S38**		
SX5	2018/TH4CN1/071804A	**S54**		
SX6	2018/TS4CN1/071808A	**S51**		
